# Microtubule-associated protein 4 phosphorylation regulates epidermal keratinocyte migration and proliferation

**DOI:** 10.7150/ijbs.35440

**Published:** 2019-07-24

**Authors:** Junhui Zhang, Lingfei Li, Qiong Zhang, Wensheng Wang, Dongxia Zhang, Jiezhi Jia, Yanling Lv, Hongping Yuan, Huapei Song, Fei Xiang, Jiongyu Hu, Yuesheng Huang

**Affiliations:** 1 Institute of Burn Research, Southwest Hospital, Third Military Medical University (Army Medical University), Chongqing, China.; 2 State Key Laboratory of Trauma, Burns and Combined Injury, Southwest Hospital, Third Military Medical University (Army Medical University), Chongqing, China.; 3 Endocrinology Department, Southwest Hospital, Third Military Medical University (Army Medical University), Chongqing, China.

**Keywords:** keratinocyte, MAP4, phosphorylation, p38/MAPK, migration, proliferation

## Abstract

Both cell migration and proliferation are indispensable parts of reepithelialization during skin wound healing, which is a complex process for which the underlying molecular mechanisms are largely unknown. Here, we identify a novel role for microtubule-associated protein 4 (MAP4), a cytosolic microtubule-binding protein that regulates microtubule dynamics through phosphorylation modification, as a critical regulator of epidermal wound repair. We showed that MAP4 phosphorylation was induced in skin wounds. In an aberrant phosphorylated MAP4 mouse model, hyperphosphorylation of MAP4 (S737 and S760) accelerated keratinocyte migration and proliferation and skin wound healing. Data from both primary cultured keratinocytes and HaCaT cells* in vitro* revealed the same results. The promigration and proproliferation effects of MAP4 phosphorylation depended on microtubule rearrangement and could be abolished by MAP4 dephosphorylation. We also identified p38/MAPK as an upstream regulator of MAP4 phosphorylation in keratinocytes. Our findings provide new insights into the molecular mechanisms underlying wound-associated keratinocyte migration and proliferation and identify potential targets for the remediation of defective wound healing.

## Introduction

Wound healing is a complex and precisely regulated process that mainly involves the following three phases: inflammation, reepithelialization and tissue remodeling. Keratinocyte proliferation and migration are crucial steps in the reepithelialization process 1. A multitude of molecular changes could enable keratinocytes to acquire high motility, undergo mitosis and subsequently heal the wound 2-4. A better understanding of these mechanisms would undoubtedly help to enhance the wound repair process.

Microtubules (MTs), critical components of the cytoskeleton, play an important role in regulating various processes, including cell migration and proliferation 5-7. Microtubule-associated protein 4 (MAP4) is a member of the microtubule-associated protein (MAP) family, has an N-terminal projection domain and a C-terminal MT-binding domain and is responsible for the stabilization of MTs by modulating MT dynamics 8-10. MAP4-MT binding ability is regulated by MAP4 phosphorylation. Once phosphorylated, MAP4 detaches from MTs, which results in MT instability. In humans, the critical phosphorylation sites responsible for MAP4 detachment from MTs are S768, S787 and S696 (the corresponding sites in mice are predicted to be S737, S760 and S667) 11-13. Recently, several studies have shown that MAP4 promotes cell invasion in cancer progression 14-16. However, the contribution of MAP4 to wound healing is still not clear nor is the regulation of MAP4 phosphorylation during wound healing.

Previously, we reported that p38/MAPK activation could lead to MAP4 phosphorylation with MT disassembly in cardiomyocytes during hypoxia 10 and in microvascular endothelial cells during inflammation 9. In addition, p38/MAPK is an important signal required for the cytoskeletal reorganization that precedes cell migration and proliferation 17, 18. Therefore, it is speculated that MAP4 phosphorylation controlled by p38/MAPK plays a role in regulating the migration and proliferation of keratinocytes by affecting MT dynamics.

In this study, we aimed to investigate the role of MAP4 phosphorylation in wound repair. We found that MAP4 phosphorylation accelerated wound healing by promoting epidermal cell migration and proliferation. We also showed that the induction of MAP4 phosphorylation in response to wound stimuli was dependent on p38/MAPK, a kinase that had previously been shown to be required for efficient wound healing. Mechanistically, MAP4 phosphorylation promoted cell migration and proliferation by modulating MT dynamics in keratinocytes. Our combined data delineate a critical role for MAP4 and MT dynamics in promoting wound healing.

## Materials and Methods

### Ethics Statement

All animal experiments were carried out according to the guidelines of the Care and Use of Laboratory Animals published by the National Institutes of Health (NIH Pub. No. 85-23, revised 1996) and authorized by the Animal Experiment Ethics Committee of the Third Military Medical University in Chongqing, China.

### Generation of the MAP4 (S667A, S737E and S760E) knockin (KI) mice

The KI mice were constructed and identified by the Shanghai Biomodel Organism Science & Technology Development Co., Ltd. Cas9 mRNA, guide RNAs and the donor vector were microinjected into fertilized eggs (C57BL/6J) before the eggs were implanted into surrogate C57BL/6J females as described in our previous study 19.

### * In vivo* wound closure assay

For wounding experiments, 8- to 12-week-old male/female KI or littermate control mice were anesthetized with intraperitoneal administration of sodium pentobarbital, and full-thickness wounds were made on the mid-dorsal skin with 5-mm disposable biopsy punches. Digital photographs were taken of the injury site, which was circumscribed and identified by standard-sized dot that was placed beside the wound. Wound size was defined as the ratio of the wound area to the dot measurement. For histological examination, skin was harvested and fixed in 4% paraformaldehyde at the indicated time points: frozen sections were used to detect the phosphorylation levels of MAP4; paraffin sections were stained with hematoxylin and eosin (HE). Rabbit anti-p-MAP4 primary antibody was used for immunofluorescence staining of p-MAP4 in wound specimens.

### Cells culture

Immortalized human keratinocyte HaCaT cells were obtained from the Cell Bank of the Chinese Academy of Sciences in Beijing, China and cultured in RPMI 1640 medium (SH30809, HyClone) supplemented with 10% fetal bovine serum (10100139, Gibco), 100 U/ml penicillin, and 100 μg/ml streptomycin (Beyotime, China). Primary mouse keratinocytes (MKs) were cultured as previously reported 20. Briefly, MKs were isolated from the epidermis of newborn C57BL/6 mice using 0.25% trypsin/0.04% EDTA solution (Invitrogen, USA) after prior separation of the epidermis from the dermis by an overnight dispase treatment (4 °C) and then cultured in keratinocyte serum-free medium (K-SFM medium) (Gibco, USA). The keratinocytes were incubated at 37 °C, 5% CO_2_, and 95% humidity.

### Hypoxia exposure

Hypoxic conditions of 2% O_2_, 5% CO_2_ and 93% N_2_ were created by a constant flow of nitrogen using a Forma Series II Water Jacket CO_2_ incubator (model: 3131; Thermo Scientific), which maintained the desired temperature (37 °C) and O_2_ level. A p38 inhibitor (SB203580, Invitrogen, 5 μM) was added to the cultures and incubated at 37 °C for 30 min prior to hypoxic treatment.

### *Ex vivo* skin explant outgrowth assay

Circular skin biopsies were obtained from the dorsal skin of neonatal mice of the same litter using 4-mm biopsy punches and were plated on 24-well plates with 200 μl of medium containing the following additives (final concentrations) 21-23: cholera toxin (0.1 nM), epidermal growth factor (10 ng/ml), transferrin (5 μg/ml), insulin (5 μg/ml), hydrocortisone (0.4 μg/ml), T3 (2 nM), penicillin (100 U/ml), and streptomycin (100 μg/ml). After incubation at 37 °C and 5% CO_2_ overnight, explants were submerged in the prepared medium. To inhibit cell proliferation, we subjected skin explants to mitomycin C (5 mg/ml, Selleck) 48 h after being plated on 24-well plates. Then, cellular outgrowth from each explant was monitored with the Zeiss imaging system (Carl Zeiss Meditec, Jena, Germany), and the proximal distance extending between the explant biopsy and the distal edge of the cellular migration was measured using NIH ImageJ software (http://rsb.info.nih.gov/ij/).

### Recombinant adenovirus construction and transduction

The recombinant adenovirus that expressed the constitutively activated mitogen-activated protein kinase 6 (MKK6(Glu)), which selectively and constitutively activates p38/MAPK signaling, was produced by Shanghai GeneChem, Co. Ltd. (Shanghai, China). The CMV-null adenovirus was used as the negative control.

### Site-directed mutagenesis of MAP4(Ala) recombinant adenovirus construction and transduction

The site-directed mutagenesis of MAP4(Ala) recombinant adenovirus was prepared using the AdMax^TM^ system (Microbix, Ontario, Canada) according to the manufacturer's instructions as reported previously 10. Primers were designed to generate the MAP4 point mutations S768A and S787A through PCRs using the QuikChange Multi Site-Directed Mutagenesis Kit (200514, Stratagene). The CMV-null adenovirus was used as the negative control.

### Extraction and quantification of tubulin fractions

The free and polymerized tubulin fractions were isolated as previously described 24. The MT stabilization buffer (MTSB, 37 °C) with 2 mM ethylene glycol-bis (β-aminoethylether)-N, N, N', N'-tetraacetic acid (EGTA), 0.1 M piperazine-N, N'-bis (2-ethanesulfonic acid, pH 6.8) (PIPES), 0.5 mM MgCl_2_, 20% glycerol and 2 mM ethylenediaminetetraacetic acid (EDTA) was used to wash the cells cultured in 6-well plates. Cells were incubated with MTSB containing 0.1% Triton X-100, protease inhibitor cocktail, phenylmethylsulfonyl fluoride, and phosphatase inhibitor cocktail (Sigma-Aldrich). Then, Western blotting was performed to analyze the polymerized and free tubulin fractions.

### Western blot analysis

Whole keratinocyte extracts and mouse skin specimens were prepared in RIPA lysis buffer (P0013, Beyotime) for Western blotting and centrifuged at 14,000 rpm for 15 min at 4 °C. The supernatants were then obtained and protein concentrations were detected using the Bradford Protein Quantification Kit (500-0205, Bio-Rad Laboratories). The protein extracts were loaded and separated by SDS-PAGE and then transferred to PVDF membranes (Millipore). The membranes were incubated overnight at 4 °C with specific primary antibodies. Then, membranes were incubated with secondary antibodies and visualized using the ChemiDoc XRS System (Bio-Rad Laboratories). The primary antibodies used for immunoblotting were as follows: p-p38 (1:1000, Cell Signaling Technology), p38 (1:1000, Cell Signaling Technology), MAP4 (1:1000, Bethyl, USA), p-MAP4(S768) (Biolegend, 1:1000), p-MAP4(S696) (1:1000, GL Biochem), p-MAP4(S787) (1:1000, GL Biochem), p-MAP4(S737) (1:1000, GL Biochem), β-Actin (1:1000, Cell Signaling Technology), PCNA (1:1000, Abcam), and Ki67 (1:1000, Abcam). The rabbit polyclonal antibodies against p-MAP4(S696), p-MAP4(S787) and p-MAP4(S737) were developed in house as described and validated in our previous report 19. Here, we validated the in-house rabbit polyclonal antibody against p-MAP4 using the amino acids QAKVG(pS)LDNVGHLPAGc and the respective nonphosphorylated peptides conjugated to bovine serum albumin (BSA) (Supplementary Fig. 1).

### Immunofluorescence staining

Cells cultured on glass coverslips were fixed in 4% paraformaldehyde for 20 min, permeabilized with 0.1% Triton X-100 in phosphate buffer saline (PBS) for 25 min, and then blocked with 10% goat serum for 1 h. To obtain the MT structure, rabbit anti-a-tubulin primary antibodies (1:100, Proteintech, USA) were diluted with PBS, and the coverslips were incubated with antibodies at 4 °C overnight. The coverslips were washed in PBS and then incubated with goat anti-rabbit secondary antibody conjugated to cyanine 3 (Cy3, 1:100) for 1 h at 37 °C. The cells were imaged using confocal microscopy (Leica Microsystems, Wetzlar, Germany).

### Cell proliferation assay

5-Ethynyl-2'-deoxyuridine (EdU) is a thymidine analog that can be incorporated to label cells undergoing DNA replication 25. Cell proliferation was determined by EdU assay carried out using the Click-iT^TM^ EdU imaging detection kit according to the manufacturer's instructions (BCK488-IV-IM-S, Sigma, USA). EdU is a thymidine analog that can be incorporated to label cells undergoing DNA replication. EdU-positive cells are defined as proliferating cells 25. Meanwhile, we used a mouse monoclonal antibody, anti-pan-keratin (1:100, Abcam), to mark keratinocytes in the regenerated epidermis. Finally, the fluorescent images were obtained by a Leica Confocal Microscope (Leica Microsystems, Germany).

### CCK-8 assay

Cell Counting Kit-8 (CCK-8, Beyotime, China) was used as previously described 26 for the detection of cell proliferation. Primary keratinocytes isolated from KI mice and WT mice were seeded in 2 × 10^3^/wells of 96-well plates and preincubated for 24 h in a humidified incubator with 5% CO_2_ at 37 °C before the CCK-8 solution (10 μl) was added to each well of the plate. Then, the cells were incubated for 1 h, and the absorbance was measured at 450 nm with a microplate reader (Thermo, USA).

### Scratch wound healing assay

Monolayers of cells plated in 12-well plates were wounded by a 10-μl plastic pipette tip after being incubated at 37 °C for 2 h with mitomycin C (S8146, Selleck, final concentration of 5 μg/ml) to inhibit cell proliferation and then rinsed with medium to remove any cell debris 27. The wound healing process was monitored with an inverted light microscope (Olympus, Japan). Cell migratory capacity was defined as the wound closure rate (%), which was analyzed using NIH ImageJ software (http://rsb.info.nih.gov/ij/).

### Single-cell motility assay and quantitative analysis

Cells were seeded into 24-well plates at a density of 0.5 × 10^4^/cm^2^ in RPMI 1640 medium. Then, time-lapse imaging was performed with a Zeiss imaging system (Carl Zeiss Meditec, Jena, Germany), which was equipped with a CO_2_- and temperature-controlled chamber. The images were acquired every 3 min for 3 h. Later, cell trajectories were obtained by tracing the position of the cell nucleus at frame intervals of 6 min using NIH ImageJ software, and the velocity (μm/min) of each cell was defined as the total length (μm) of the trajectory divided by time (min), which reflected cell motility.

### Statistical analysis

All data are expressed as the means ± SEM. Comparisons between two groups were performed using a two-tailed unpaired t-test. Statistical significance among three or more groups was performed by a one-way analysis of variance (ANOVA). *P < 0.05* was considered to be significant.

## Results

### MAP4 phosphorylation is induced at the wound edge and promotes wound repair

**Figure 1:**
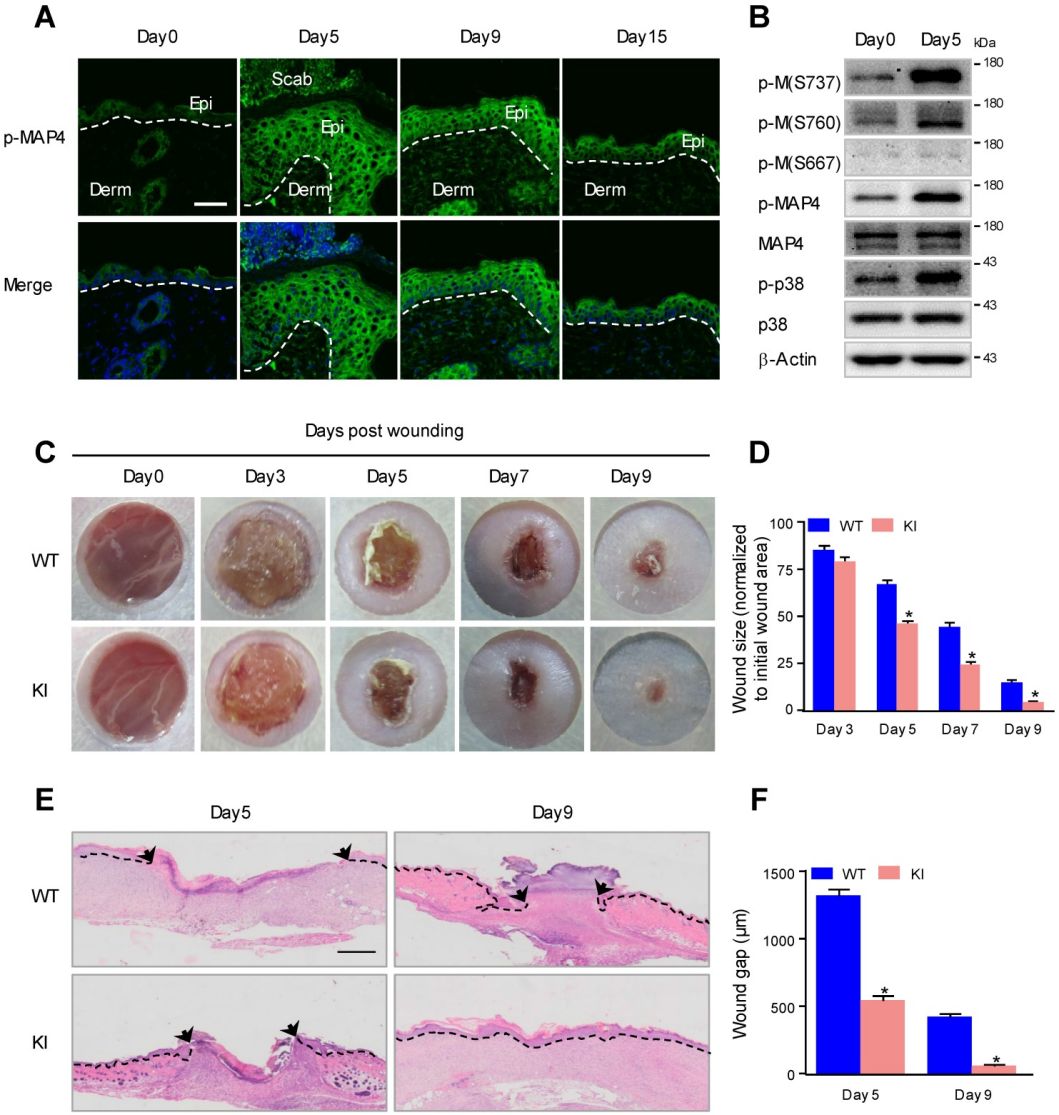
** MAP4 phosphorylation is induced at the wound edge and promotes wound repair. (A)** Immunofluorescence staining of p-MAP4 in normal unwounded skin (day 0), day 5, day 9, and day 15 wound sections obtained from wild-type (WT) C57BL/6J mice. Wounds were close to reepithelialization on day 9 and fully re-epithelialized on day 15 (n = 10). Nuclei were stained with 4',6-diamidino-2-phenylindole (DAPI, blue). Scale bar = 50 μm. Narrow dotted line: interface between epidermis and dermis or leading edge of migrating epidermis (day 5 and day 9). Epi, epidermis; Derm, dermis. **(B)** Western blotting was performed to detect the phosphorylation of MAP4 at S737, S760 and S667 in mouse epidermis as well as the activation of p38/MAPK. β-Actin was used as a loading control (n = 10). **(C)** Images of skin wound sites taken 0, 3, 5, 7, and 9 days post wounding. Full-thickness excisional wounds (5 mm in diameter) were made on dorsal skin of KI mice and their corresponding WT littermates (n = 10). **(D)** Graphs showing the rate of wound closure. Areas around the wounds were measured with ImageJ software. The results are shown as the means ± SEM. ^*^P < 0.05 vs. the corresponding WT group. **(E)** Wound healing was monitored by histological staining of skin sections (day 5 and day 9 post injury) at the wound edge. Scale bar = 200 μm. Dotted lines indicate dermal-epidermal boundaries. Arrows denote the leading edges of the epidermis (n = 10). **(F)** Graph shows the average wound gap quantified at the indicated time after wounding. The data are shown as the means ± SEM. ^*^P < 0.05 vs. the corresponding WT group.

Considering the potential role of MAP4 phosphorylation in the reepithelialization process, we first examined the level of MAP4 phosphorylation at the wound margin following full-thickness skin wounding (5 mm in diameter) in 8- to 12-week-old mice. MAP4 phosphorylation was markedly upregulated at the wound margin compared with the normal epidermis and then fell, with the maximal expression level measured at day 5 (Fig. 1A). When reepithelialization was already fully completed at day 15, MAP4 phosphorylation was still higher than that observed in normal skin epidermis (Fig. 1A), which indicated a role of MAP4 phosphorylation in wound remodeling.

The amino acid residues S768, S787 and S696 in human MAP4 have been proven to be the critical sites responsible for MAP4 binding to tubulin; phosphorylation of these sites leads to MAP4 dissociation from tubulin 9, 10. Western blots were used to further determine the phosphorylation of these sites during wound healing. As shown in Fig. 1B, MAP4 showed weak basal levels of phosphorylation at S737, S760 and S667 in normal mouse epidermis, and wounding induced a robust phosphorylation at S737 and S760, with the phosphor-MAP4 (p-MAP4) levels elevated, but p-MAP4 (S667) and the MAP4 levels remained unchanged.

To assess the potential effect of elevated MAP4 phosphorylation on epidermal wound healing *in vivo*, we constructed a mutant KI mouse model that mimicked MAP4 phosphorylation at specific sites (S737 and S760) (Supplementary Fig. 2) 19. Macroscopically, the wound closure area, quantified at different time points following wounding, showed that, from day 5 post wounding, healing was significantly accelerated in the KI group compared with the wild-type (WT) group (Fig. 1C and D). Histologically, the results of wound morphology revealed that the wound gap remained significantly narrower in KI mice than in the WT group from day 5 to day 9 after wounding. At day 9, the wounds in KI mice were completely re-epithelialized, while the epithelium layer in WT mice remained open (Fig. 1E and F). These results demonstrated that elevated MAP4 phosphorylation promoted wound healing and strongly suggested a critical role for MAP4 phosphorylation in wound repair* in vivo*.

### MAP4 phosphorylation promotes the proliferation of epidermal keratinocytes

**Figure 2:**
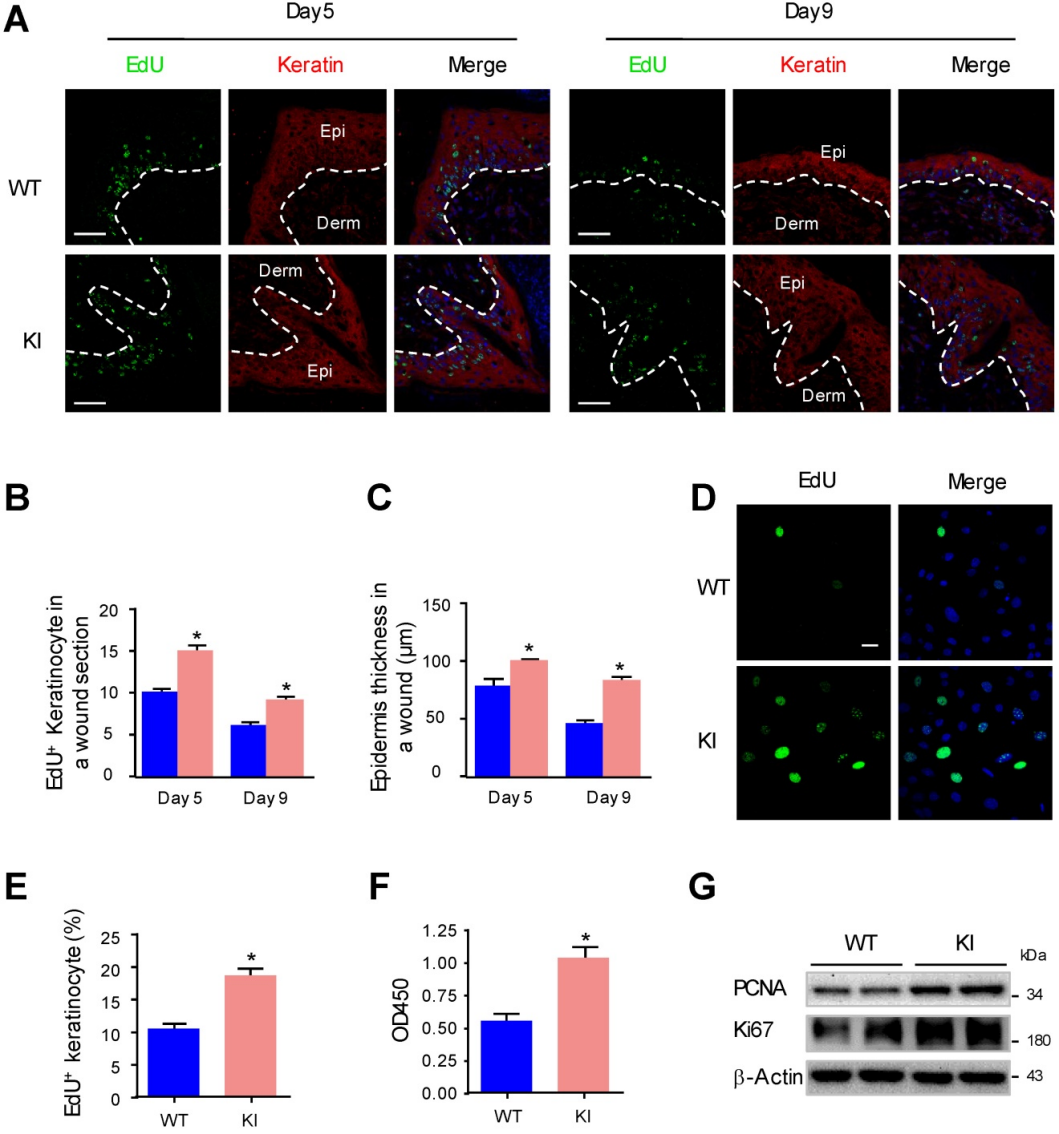
** MAP4 phosphorylation promotes the proliferation of epidermal keratinocytes. (A)** Representative pictures of confocal images of EdU staining (green) and pankeratin (red) of WT and KI mice wounds on day 5 and day 9 after injury (n = 10). Nuclei were stained with DAPI (blue). Narrow dotted line: interface between epidermis and dermis or leading edge of migrating epidermis. Scale bar = 50 μm. **(B)** Quantification of the EdU-positive keratinocytes at times indicated after wounding (A). The data are shown as the means ± SEM. ^*^P < 0.05 vs. the corresponding WT group. **(C)** Quantification of the average epidermal thickness at times indicated after wounding (A). The data are shown as the means ± SEM. ^*^P < 0.05 vs. the corresponding WT group. **(D)** Representative pictures of EdU staining (green) of MKs isolated from the epidermis of KI and WT mice (n = 5). Nuclei were stained with DAPI (blue).** (E)** Graph shows quantification data of the EdU-positive keratinocytes shown in (D). The data are shown as the means ± SEM. ^*^P < 0.05 vs. the corresponding WT group. **(F)** Keratinocyte proliferation was evaluated using a CCK-8 assay according to manufacturer's instructions. The results are shown as means ± SEM (n = 5).^ *^P < 0.05 versus the WT group. **(G)** Western blotting was performed to analyze the expression of PCNA and Ki67 in cultured keratinocytes isolated from the epidermis of KI and WT mice (n = 5). Representative bands of two samples in each group are shown. β-Actin was used as a loading control.

Keratinocyte proliferation has been proven to be important for reepithelialization during wound healing 28. To further investigate whether MAP4 phosphorylation plays a direct role in epidermal keratinocyte proliferation, we compared the proliferation of keratinocytes at the wound edge in KI mice and WT mice by performing EdU staining. The proliferating keratinocytes are indicated as EdU (+) and keratin (+). The results showed that the proliferation rate of keratinocytes at the wound edge in the KI group was increased compared to that in the WT group (Fig. 2A and B). Analysis of epidermal thickness showed a marked thickening of the epithelium in the KI group compared with the WT group (Fig. 2C). Furthermore, we performed proliferation assays with primary keratinocytes isolated from KI mice and WT mice. The results of the EdU staining and CCK-8 assay demonstrated that, compared with WT controls, KI keratinocytes showed a significant increase in cell proliferation (Fig. 2D-F). Western blots revealed that levels of PCNA and Ki67 in KI keratinocytes were much higher compared with the WT group (Fig. 2G), which indicated that KI keratinocytes grew much faster than WT keratinocytes. These results suggested that MAP4 phosphorylation promoted keratinocyte proliferation.

### MAP4 phosphorylation promotes epidermal keratinocyte migration and regulates MT rearrangement

**Figure 3:**
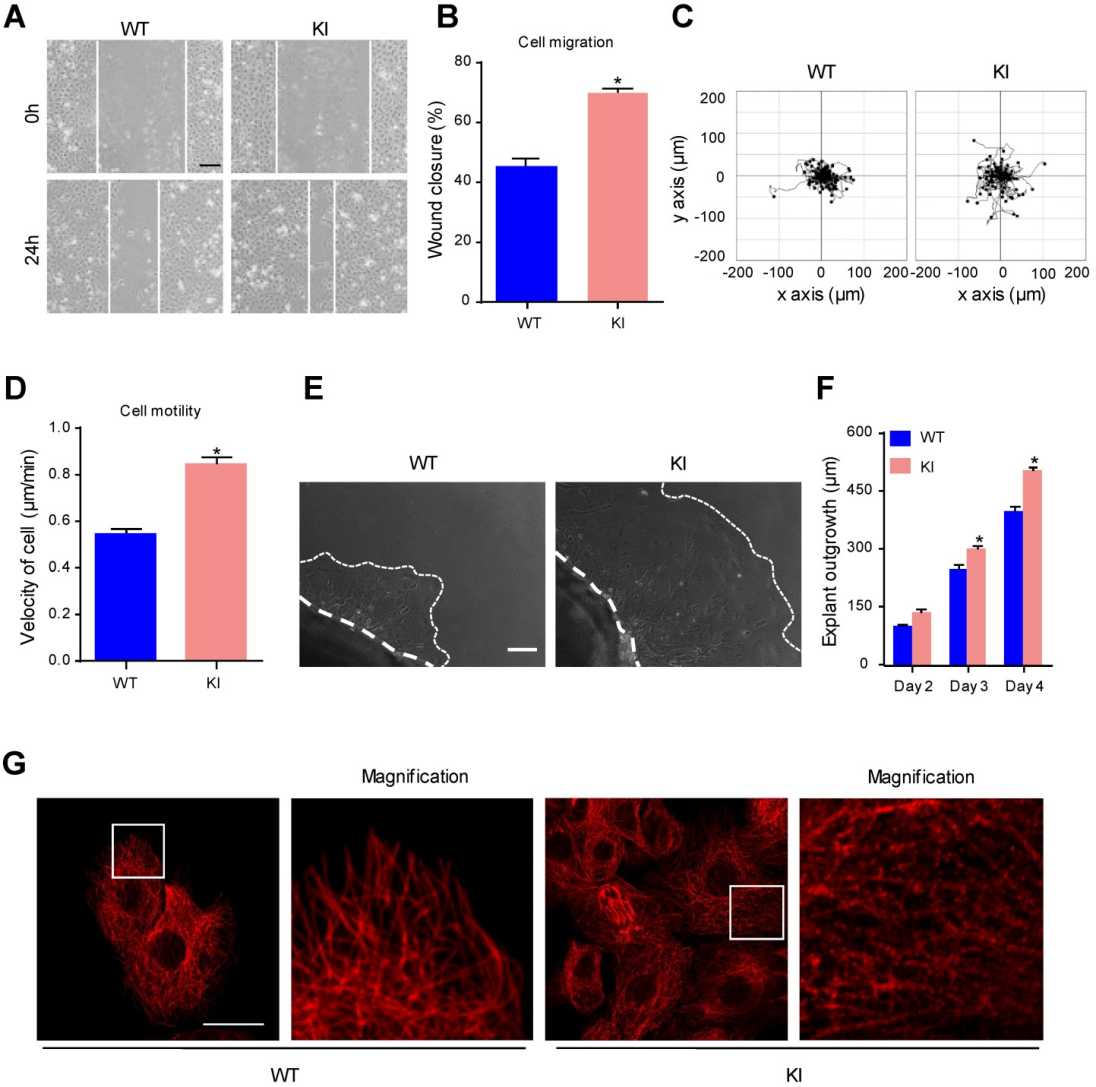
** MAP4 phosphorylation promotes epidermal keratinocyte migration and regulates MT rearrangement.** Scratch wound healing assays **(A, B)** and single-cell motility assays **(C, D)** were performed using MKs isolated from the epidermis of KI and WT mice (n = 5). Representative pictures of wound healing and the trajectories of keratinocytes are shown. Scale bar = 100 μm. Quantitative results are shown as the means ± SEM (n = 5). ^*^P < 0.05 vs. WT group. **(E)** Representative images of skin explant culture (day 4). Dotted lines denote the boundary of skin explant (left) or leading edge of epidermal outgrowth from the explant (right) (n = 10). Scale bar = 100 μm. **(F)** Quantification of the outgrowth of epidermal explants. The data are shown as the means ± SEM. ^*^P < 0.05 vs. the corresponding WT group. **(G)** Staining of MTs in the indicated keratinocytes (n = 5). The boxed areas show image at higher magnification to illustrate details. Scale bar = 25 μm. All experiments were repeated 3 times.

During the process of reepithelialization in wound healing, epidermal cells migrate into the wound site, proliferate and differentiate to regenerate the epidermal barrier 29. To explore whether MAP4 phosphorylation is involved in cell migration, we measured cell migration using a scratch wound healing assay and a single-cell motility assay with primary keratinocytes isolated from KI mice and WT mice. We found that keratinocytes in the KI group showed a significant increase in cell migration compared with keratinocytes in the WT group (Fig. 3A and B). The results of the single-cell motility assay showed a remarkable increase in the range of cell movement and the cell velocity in the KI group (Fig. 3C and D). Using explant skin culture *ex vivo*, we also found that epithelial cells from KI skin migrated more quickly than cells from WT control skin (Fig. 3E and F). Together, these findings demonstrated that MAP4 phosphorylation could promote keratinocyte migration.

MT disruption facilitates the assembly of focal adhesions and enhances cell migration 6. As a cytosolic MT-binding protein, MAP4 has been proven to play an important role in MT dynamics. Once phosphorylated, MAP4 dissociates from tubulin, resulting in MT instability 10. Western blots showed reduced levels of polymerized tubulin and increased the levels of free tubulin in keratinocytes from the KI group (Supplementary Fig. 6A). In the morphological studies, keratinocytes in the KI group showed clear signs of MT disruption and modification (Fig. 3G), suggesting that MAP4 phosphorylation-modified MTs may contribute to the proliferation and migration of keratinocytes by inducing MT depolymerization.

### MAP4 phosphorylation regulates hypoxia-induced epidermal keratinocyte proliferation and migration through MT rearrangement

**Figure 4:**
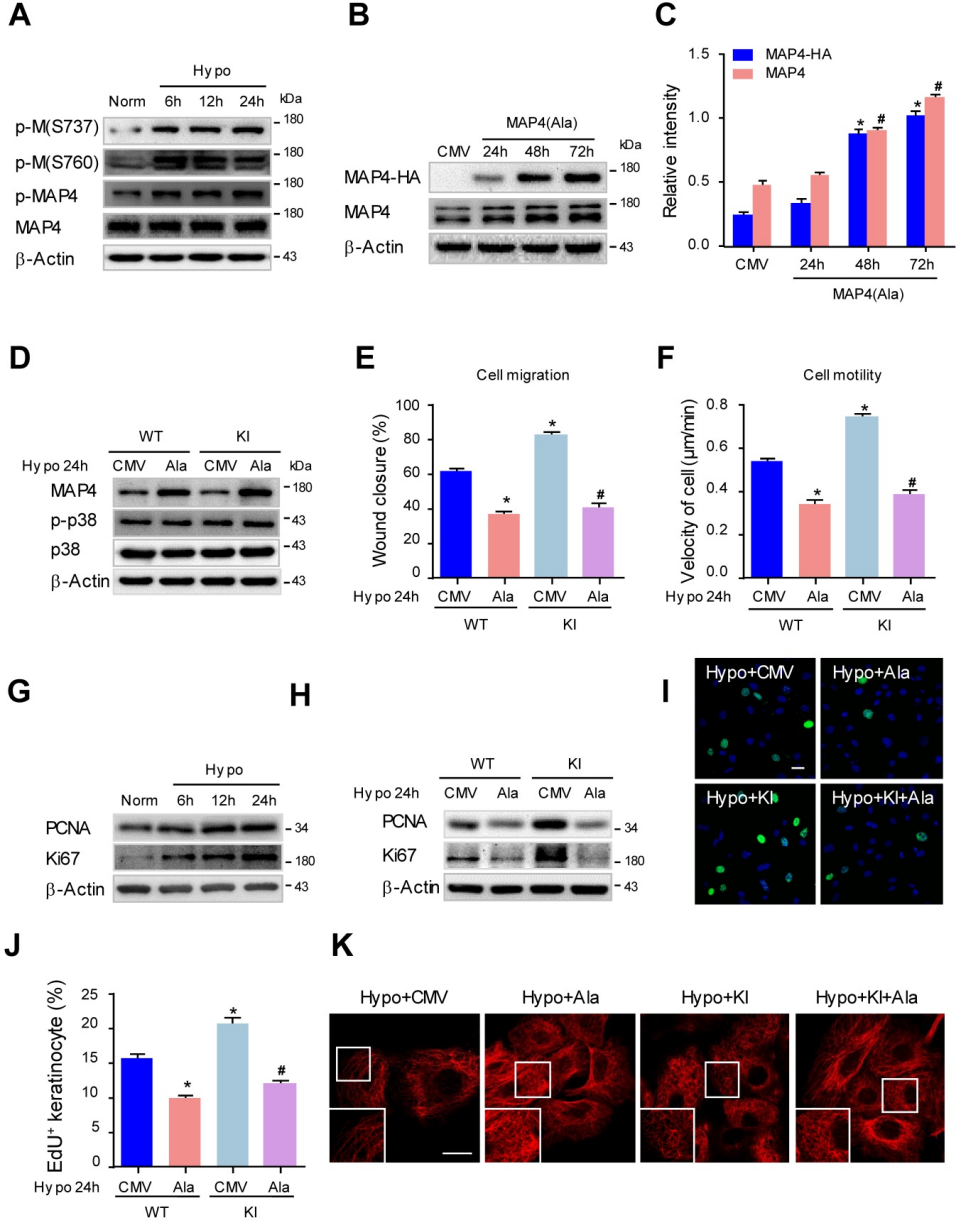
** MAP4 phosphorylation regulates hypoxia-induced epidermal keratinocyte proliferation and migration through MT rearrangement. (A)** MKs were exposed to hypoxia (2% O_2_) and incubated for the indicated times, and total proteins were harvested for detection of MAP4 phosphorylation by Western blotting. β-Actin was used as a loading control (n = 5).** (B)** Confirmation of adenovirus transfection at comparable levels in MKs. Total cell extracts from MKs after transfecting MAP4(Ala) or CMV-null adenovirus were analyzed by Western blotting (n = 5). **(C)** Graphs indicate the relative intensities as determined by Quantity one software. Results are shown as the means ± SEM. ^*/#^ *P* < 0.05 vs. the corresponding CMV-null (CMV) group.** (D)** MKs isolated from the epidermis of KI or WT mice were subjected to hypoxia (2% O_2_, 24 h) after being transfected with CMV-null or MAP4 (Ala) for 48 h. The Western blot shows activation of p38/MAPK (n = 5); β-Actin was used as the loading control. Then, cell migration and motility were assessed by scratch wound healing assays **(E)** and single-cell motility assays **(F)**. The quantitative results are shown as the means ± SEM. ^*^P < 0.05 vs. Hypo + WT + CMV group, ^#^*P* < 0.05 vs. Hypo + KI + CMV group (n = 5). **(G)** MKs were exposed to hypoxia (2% O_2_) and incubated for the indicated times, and total proteins were harvested for detection of the expression of PCNA and Ki67 using Western blotting (n = 5). Representative bands are shown. β-Actin was used as a loading control. **(H)** MKs isolated from the epidermis of KI and WT mice were subjected to hypoxia (2% O_2_, 24 h) after being transfected with CMV-null or MAP4(Ala) for 48 h. Representative blots show the expression of PCNA and Ki67 (n = 5); β-Actin was used as the loading control. **(I)** Representative pictures of EdU staining (green) of the indicated keratinocytes. Nuclei were stained with DAPI (blue). Scale bar = 50 μm (n = 5). (**J**) Quantification of the positive rate of EdU in indicated keratinocytes. The results are shown as the means ± SEM. ^*^P < 0.05 vs. Hypo + WT + CMV group, ^#^*P* < 0.05 vs. Hypo + KI + CMV group.** (K)** MTs stained in the indicated keratinocytes. The boxed areas show higher magnification to illustrate details (n = 5). Scale bar = 25 μm. All experiments were repeated 3 times. Hypo, hypoxia.

Previously, we and others determined that hypoxia promoted keratinocyte migration in wound repair 27, 30, and as a consequence, hypoxia induced MAP4 phosphorylation with MT depolymerization in cardiomyocytes 10. To investigate whether MAP4 phosphorylation contributed to hypoxia-induced keratinocyte migration and proliferation in wound repair, MAP4 phosphorylation was analyzed by Western blotting of cultured primary keratinocytes with or without hypoxic treatment. As shown in Fig. 4A, MAP4 showed low basal levels of phosphorylation at S737 and S760 in cultured epidermal keratinocytes under normoxic conditions. Hypoxia (2% O_2_) induced elevated phosphorylation at both residues and p-MAP4 in a time-dependent manner, with the MAP4 levels unchanged.

To confirm whether the phosphorylation of MAP4 indeed regulated keratinocyte migration and proliferation, we constructed adenoviruses overexpressing the dephosphorylated form of MAP4 by changing S768 and S787 to alanine (MAP4(Ala)). HA-tagged MAP4(Ala) and CMV-null were overexpressed at comparable levels in epidermal keratinocytes as verified by Western blot analysis (Fig. 4B, and C). Then, we transfected MAP4(Ala) or CMV-null adenovirus into cultured keratinocytes isolated from KI or WT mice (Fig. 4D) and detected changes in cell migration and proliferation. As expected, the increased migratory capacity of keratinocytes under hypoxic stress (2% O_2_, 24 h) in both the WT and KI group was dramatically restored by MAP4(Ala) transfectants compared with cells transfected with CMV-null, as assessed by the wound healing assay or single-cell motility assay (Fig. 4E-F, supplementary Fig. 3). Similarly, the proliferation of keratinocytes induced by hypoxia was markedly reduced after MAP4(Ala) transfection compared with that in cells transfected with CMV-null, as depicted by EdU staining or expression of PCNA and Ki67 (Fig. 4G-J).

The effect of MAP4(Ala) overexpression on the MT arrangement in keratinocytes was first observed using confocal microscopy (Fig. 4K). Hypoxia (2% O_2_ for 24 h) resulted in significant MT depolymerization represented by a thin and disrupted residual network in keratinocytes. MAP4(Ala) transfection alleviated the hypoxia-induced MT depolymerization in both the WT and KI. Similarly, Western blots showed that MAP4(Ala) transfection reversed the reduced levels of polymerized tubulin and increased the levels of free tubulin in hypoxic cells from the WT and KI groups (Supplementary Fig. 6B). Collectively, these data suggested that the phosphorylation of MAP4 at S737 and S760 might promote cell migration and proliferation under hypoxia by inducing MT depolymerization in epidermal keratinocytes.

### P38/MAPK is involved in MAP4 phosphorylation-induced epidermal keratinocyte migration and proliferation under hypoxia

**Figure 5:**
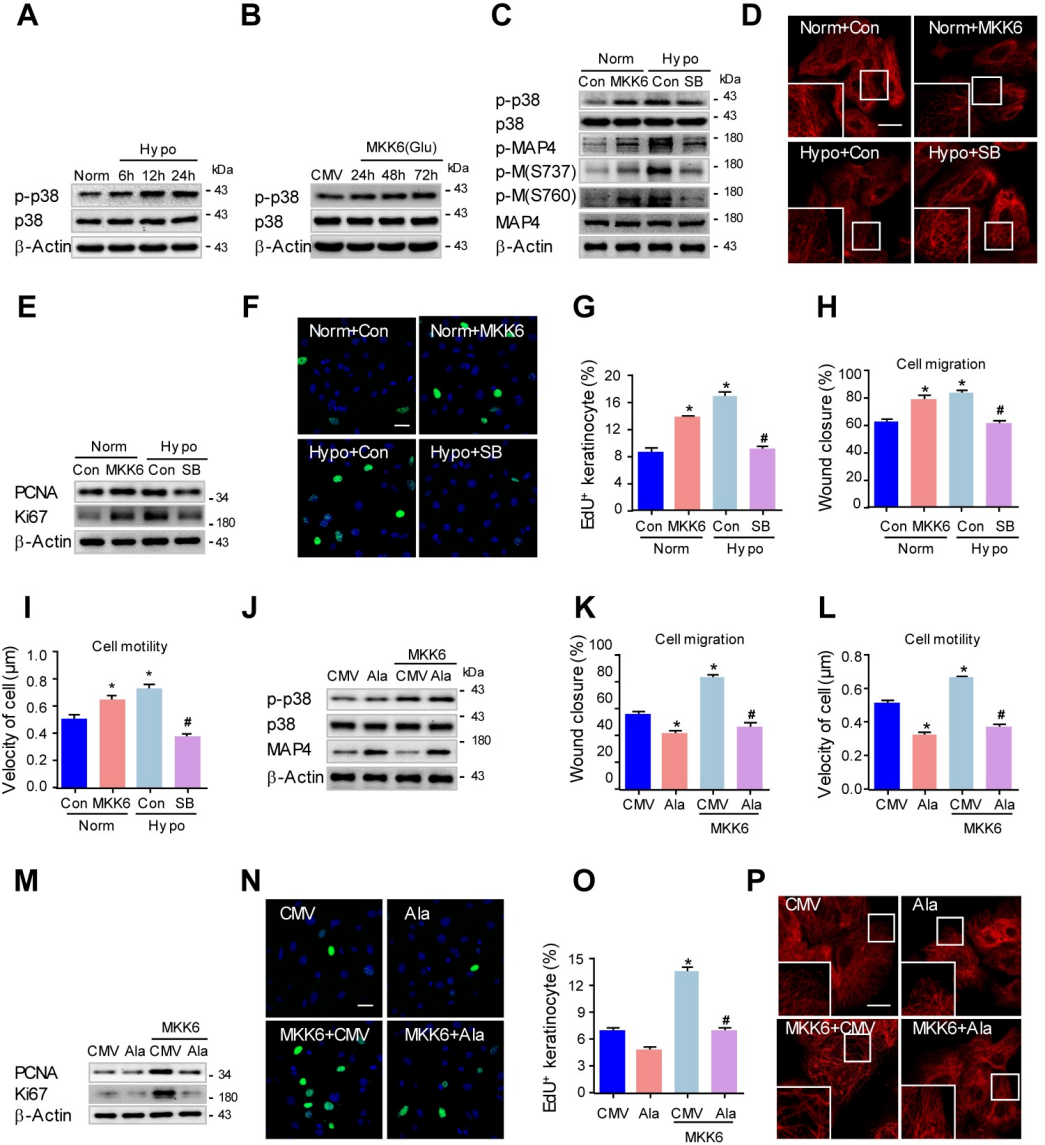
** P38/MAPK is involved in MAP4 phosphorylation-induced epidermal keratinocyte migration and proliferation under hypoxia. (A)** MKs were exposed to hypoxia (2% O_2_) and incubated for the indicated times, and cell proteins were harvested for detection of activated of p38/MAPK using Western blotting. β-Actin was used as a loading control (n = 5).** (B)** Confirmation of adenovirus transfection at comparable levels in MKs. Cell extracts from MKs after transfection with MKK6(Glu) adenovirus were analyzed by Western blotting (n = 5).** (C)** MKs were transfected with MKK6(Glu) adenovirus under normoxia or subjected to a specific p38/MAPK inhibitor SB203580 (SB, 5 μM) before hypoxia exposure (2% O_2_, 24 h). Western blot analysis showed the activities of p38/MAPK and MAP4 phosphorylation of MKs with the indicated treatments (n = 5). β-Actin was used as the loading control. **(D)** MTs stained in the indicated keratinocytes (n = 5). The boxed areas show higher magnification to illustrate details. Scale bar = 25 μm.** (E)** Western blotting was performed to analyze the expression of PCNA and Ki67 in the indicated keratinocytes (n = 5). **(F)** Representative pictures of EdU staining (green) of the indicated keratinocytes (n = 5). Nuclei were stained with DAPI (blue), scale bar = 50 μm.** (G)** Graphs indicate the positive rate of EdU in the indicated MKs. Results are shown as the means ± SEM. ^*^P < 0.05 vs. Norm + Con group, ^#^*P* < 0.05 vs. Hypo + Con group. Then, scratch wound healing assays **(H)** and single-cell motility assays **(I)** were performed to determine the migration of indicated keratinocytes, and the quantitative results are shown as the means ± SEM (n = 5). ^*^P < 0.05 versus the Norm + Con group, ^#^*P* < 0.05 versus the Hypo + Con group.** (J)** Western blotting was performed to analyze the activities of p38/MAPK and the expression of MAP4 in MKs transiently transfected with MAP4(Ala), MKK6(Glu) or both (n = 5). β-Actin was used as the loading control. Then, scratch wound healing assays **(K)** and single-cell motility assays **(L)** were performed to determine the migration of the indicated keratinocytes. The quantitative results are shown as the mean ± SEM. ^*^P < 0.05 vs. CMV group, ^#^*P* < 0.05 vs. MKK6 + CMV group. **(M)** Western blotting was performed to analyze the expression of PCNA and Ki67 in the indicated keratinocytes (n = 5). **(N)** Representative pictures of EdU staining (green) in the indicated keratinocytes (n = 5). Nuclei were stained with DAPI (blue). Scale bar = 50 μm. **(O)** Graphs show the positive rate of EdU in the indicated MKs. The results are shown as the means ± SEM. ^*^P < 0.05 vs. CMV group, ^#^*P* < 0.05 vs. MKK6 + CMV group. **(P)** MTs stained in the indicated keratinocytes (n = 5). The boxed areas show higher magnification to illustrate details. Scale bar = 25 μm. All experiments were repeated 3 times. Con, control.

Considering that p38/MAPK was the upstream kinase of MAP4 phosphorylation in hypoxic cardiomyocytes 10, and p38/MAPK activation and MAP4 phosphorylation were consistently increased in the epidermis at the wound edge (Fig. 1B), there is already enough reason to doubt that p38/MAPK signaling was involved in hypoxia-induced MAP4 phosphorylation and MT disassembly. We first investigated the activation of p38/MAPK under hypoxia (2% O_2_) in epidermal keratinocytes and found that p38/MAPK was significantly activated from 6 h to 24 h after hypoxic treatment (2% O_2_) (Fig. 5A).

We then investigated whether p38/MAPK activation was essential for MAP4 phosphorylation. MKK6(Glu), the persistently activated mutant of MKK6, was used to activate p38/MAPK (Fig. 5B), and a specific p38/MAPK inhibitor SB203580 (5 μM) was used to inhibit p38/MAPK activation in epidermal keratinocytes under hypoxic treatment (2% O_2_). Western blots demonstrated that the MAP4 phosphorylation at both S737 and S760 in the MKK6(Glu) group increased significantly, and SB203580 (5 μM) decreased the MAP4 phosphorylation induced by hypoxic treatment (Fig. 5C). Additionally, MKK6(Glu) induced MT disassembly, while SB203580 was found to protect against MT depolymerization under hypoxia, as assessed by confocal microscopy (Fig. 5D) and Western blots (Supplementary Fig. 6C). Then, we detected the influence of p38/MAPK activation on keratinocyte proliferation and migration. MKK6(Glu) was found to promote keratinocyte proliferation under normoxia, while SB203580 (5 μM) was verified to inhibit keratinocyte proliferation under hypoxia, as detected by detecting the PCNA and Ki67 expression, as well as EdU staining (Fig. 5E-G). On the other hand, MKK6(Glu) induced keratinocyte migration and motility under normoxia, while SB203580 (5 μM) inhibited keratinocyte migration under hypoxia, as assessed by the wound healing assay and the single-cell motility assay (Fig. 5H and I; supplementary Fig. 4A and B). These results suggested that p38/MAPK was a pivotal upstream kinase of MAP4 phosphorylation and promoted MT depolymerization, proliferation and migration in primary keratinocytes under hypoxia.

Furthermore, to clarify the role of MAP4 phosphorylation in p38/MAPK-induced keratinocyte proliferation and migration, keratinocytes were transiently transfected with MAP4(Ala), MKK6(Glu) or both. MKK6(Glu)-induced p38/MAPK activation was not influenced by MAP4(Ala) cotransfection (Fig. 5J), which further proved that p38/MAPK acted as the upstream activator of MAP4 phosphorylation. As expected, MAP4(Ala) overexpression abolished MKK6(Glu)-induced migration (Fig. 5K and L; supplementary Fig. 4C and D) and proliferation (Fig. 5M-O). In addition, MAP4(Ala) overexpression abrogated the MKK6(Glu)-induced MT disassembly (Fig. 5P, supplementary Fig. 6E). Taken together, these observations further suggested a critical role of MAP4 phosphorylation in keratinocyte migration and proliferation as the downstream effector of p38/MAPK.

### MAP4 phosphorylation induced by p38/MAPK promotes proliferation and migration in human keratinocytes under hypoxia

**Figure 6:**
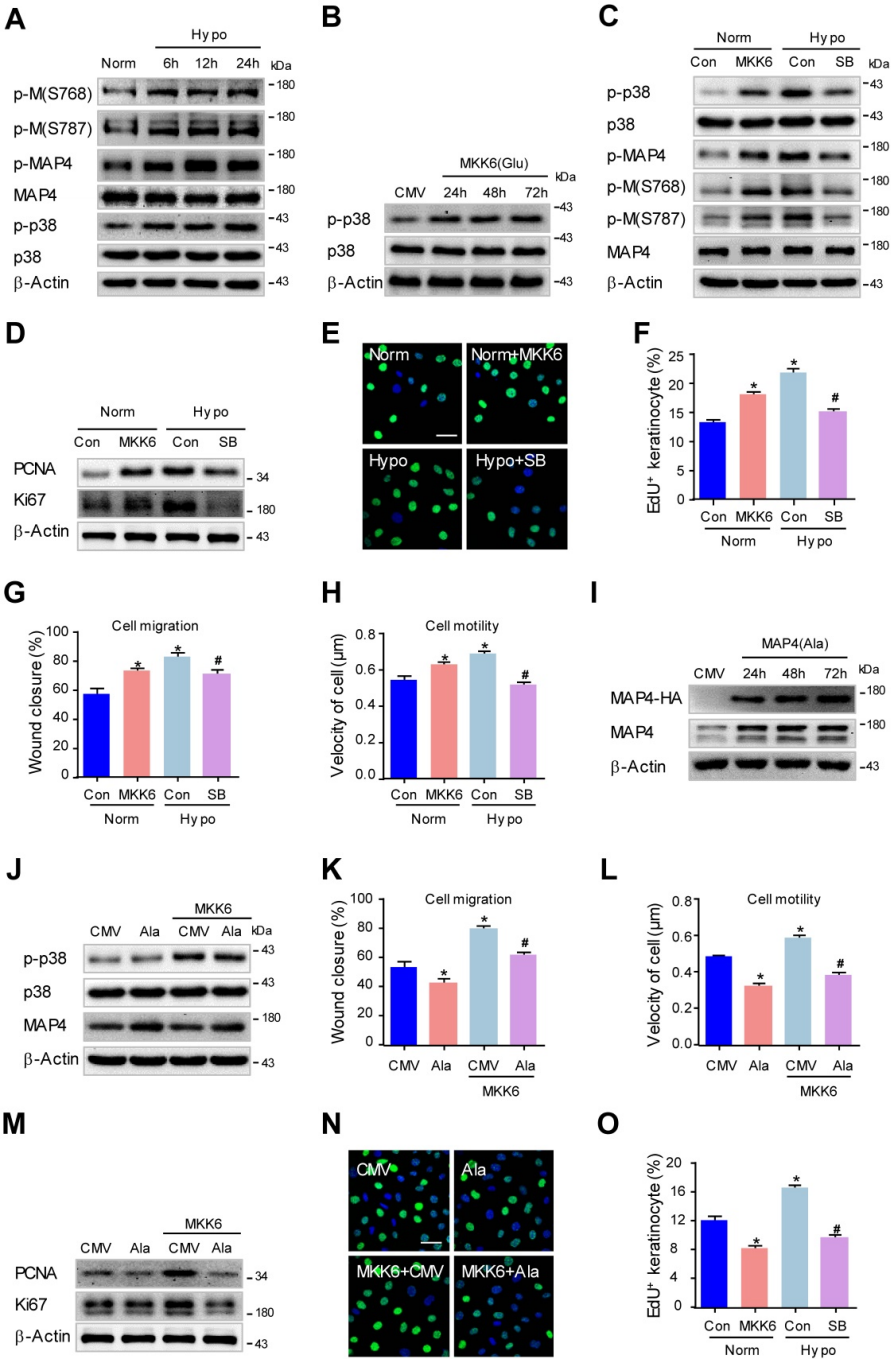
**MAP4 phosphorylation induced by p38/MAPK promotes proliferation and migration in human keratinocytes under hypoxia. (A)** HaCaT cells were exposed to hypoxia (2% O_2_) and incubated for the indicated times, and total proteins were harvested for detection of MAP4 phosphorylation and the activities of p38/MAPK using Western blotting (n = 5). β-Actin was used as a loading control.** (B)** Confirmation of adenovirus transfection at comparable levels in HaCaT cells. Cell extracts from HaCaT cells after transfection by MKK6(Glu) adenovirus were analyzed by Western blotting (n = 5).** (C)** HaCaT cells were transfected with MKK6(Glu) adenovirus under normoxia or subjected to SB (5 μM) before hypoxia exposure (2% O_2_, 24 h). The Western blot showed the activities of p38/MAPK and MAP4 phosphorylation with the indicated treatments (n = 5). β-Actin was used as the loading control. **(D)** The expression levels of PCNA and Ki67 in the indicated HaCaT cells were analyzed using Western blotting (n = 5). **(E)** Representative pictures of EdU staining (green) of the indicated keratinocytes (n = 5). Nuclei were stained with DAPI (blue). Scale bar = 50 μm. **(F)** Graphs show the positive rate of EdU in the indicated MKs (n = 5). The results are shown as the means ± SEM. ^*^P < 0.05 vs. Norm + Con group, ^#^*P* < 0.05 vs. Hypo + Con group. Then, scratch wound healing assays **(G)** and single-cell motility assays **(H)** were performed to determine the migration of the indicated keratinocytes. The quantitative results are shown as the means ± SEM (n = 5). ^*^P < 0.05 versus the Norm + Con group, ^#^*P* < 0.05 versus the Hypo + Con group. **(I)** Confirmation of adenovirus transfection at comparable levels in HaCaT cells. Cell extracts from HaCaT cells after transfection by MAP4 (Ala) adenovirus were analyzed by Western blotting (n = 5).** (J)** Western blotting was performed to analyze the activities of p38/MAPK and the expression of MAP4 in HaCaT cells transiently transfected with MAP4(Ala), MKK6(Glu) or both (n = 5). β-Actin was used as the loading control. Then, scratch wound healing assays **(K)** and single-cell motility assays **(L)** were performed to determine the migration of the indicated keratinocytes. The quantitative results are shown as the means ± SEM (n = 5). ^*^P < 0.05 vs. CMV group, ^#^*P* < 0.05 vs. MKK6 + CMV group.** (M)** The expression of PCNA and Ki67 in the indicated HaCaT cells was detected using Western blotting (n = 5). (N) Representative pictures of EdU staining (green) of the indicated keratinocytes. Nuclei were stained with DAPI (blue). Scale bar = 50 μm. **(O)** Graphs show the positive rate of EdU in the indicated MKs (n = 5). The results were shown as the means ± SEM.^ *^P < 0.05 vs. CMV group, ^#^*P* < 0.05 vs. MKK6 + CMV group. All experiments were repeated 3 times.

Considering the widely known differences in physiology and pathology between mouse and human keratinocytes, we set out to confirm the above hypothesis with immortalized human HaCaT cells. To this end, we first observed MAP4 phosphorylation and p38/MAPK activation in HaCaT cells after hypoxic treatment (2% O_2_) using Western blotting. As shown in Fig. 6A, both MAP4 and p38/MAPK showed low basal levels of phosphorylation in HaCaT cells under normoxic conditions, and hypoxic treatment (2% O_2_) induced a marked phosphorylation of both MAP4 and p38/MAPK in a consistent, time-dependent manner, with MAP4 and p38/MAPK levels unchanged. These data suggested a potential role for p38/MAPK signaling in regulating MAP4 phosphorylation in HaCaT cells under hypoxic conditions.

We then determined whether the activation of p38/MAPK was critical for MAP4 phosphorylation in HaCaT cells. MKK6(Glu) was overexpressed to activate p38/MAPK, and SB203580 (5 μM) was used to inhibit p38/MAPK activation in HaCaT cells (Fig. 6B and C). Our results showed that MKK6(Glu) stimulated MAP4 phosphorylation at S768 and S787 significantly, and the MAP4 phosphorylation induced by hypoxia was reduced by SB203580 (5 μM) treatment in HaCaT cells (Fig. 6C). Moreover, MKK6(Glu) was confirmed to promote proliferation, as detected by the expression of PCNA and Ki67 using Western blot analysis (Fig. 6D) and by EdU staining using confocal microscopy (Fig. 6E and F), as well as migration of HaCaT cells (Fig. 6G and H; supplementary Fig. 5A and B). SB203580 significantly suppressed the hypoxia-induced proliferation and migration of HaCaT cells (Fig. 6D-H; supplementary Fig. 5A and B). These results demonstrated that p38/MAPK acts as the upstream activator of MAP4 phosphorylation and promotes the proliferation and migration of HaCaT cells under hypoxia.

Next, we investigated the essential role of MAP4 phosphorylation in regulating p38/MAPK-mediated cell proliferation and migration in HaCaT cells. HA-tagged MAP4(Ala) or CMV-null was overexpressed at comparable levels in HaCaT cells and analyzed by Western blotting (Fig. 6I). Then, HaCaT cells were transiently transfected with MAP4(Ala), MKK6(Glu) or both. MKK6(Glu) significantly induced p38/MAPK activation, which was not influenced by MAP4(Ala) cotransfection, as depicted by Western blot (Fig. 6J), further proving that p38/MAPK acts as the upstream activator of MAP4 phosphorylation, as indicated by the above observations. As expected, MKK6(Glu) was found to promote proliferation and migration of HaCaT cells significantly, while MAP4(Ala) overexpression abolished p38/MAPK-induced migration (Fig. 6K and L; supplementary Fig. 5C and D) and proliferation (Fig. 6M-O) of HaCaT cells.

Moreover, we investigated the role of p38/MAPK-induced MAP4 phosphorylation in MT rearrangement in HaCaT cells. The Western blots showed that MKK6(Glu) induced MT disassembly, while the SB203580 protected against MT depolymerization under hypoxia (supplementary Fig. 6D); MAP4(Ala) overexpression abrogated the MKK6(Glu)-induced MT disassembly (supplementary Fig. 6F). These observations have, once again, proven the critical role of MAP4 phosphorylation in p38/MAPK-mediated cell proliferation and migration in HaCaT cells, which has been verified in primary mouse epidermal keratinocytes.

## Discussion

**Figure 7:**
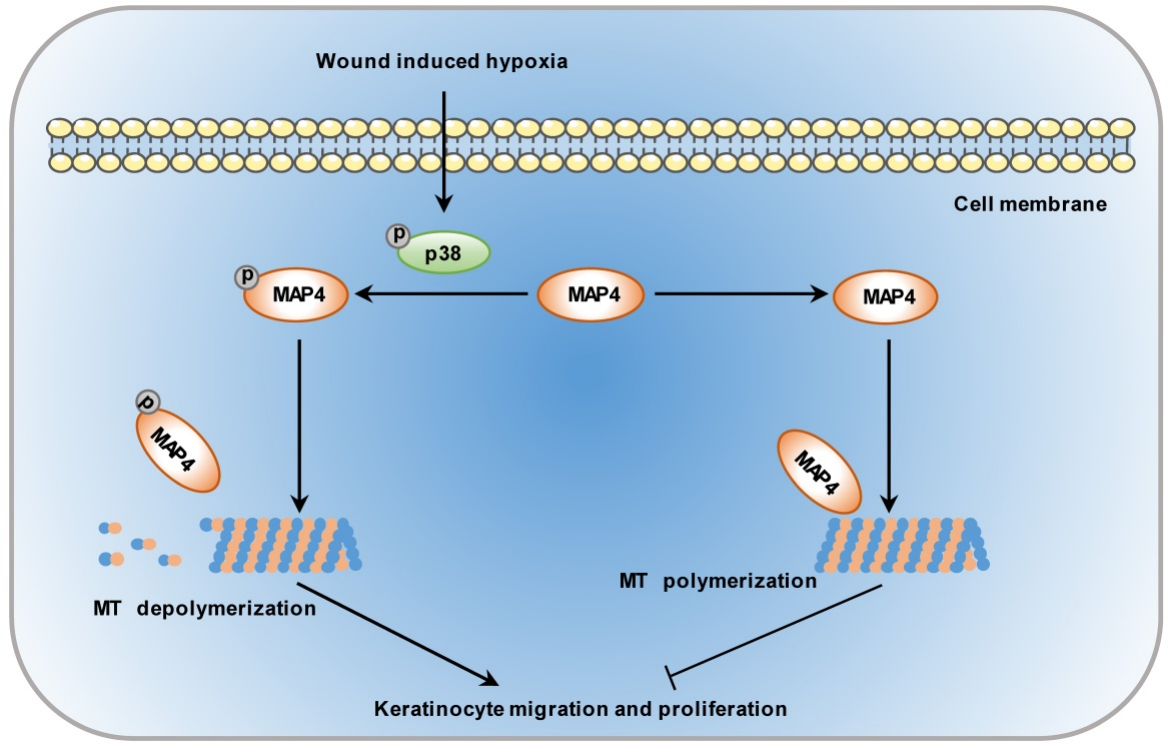
** Schematic illustrating that MAP4 phosphorylation is involved in keratinocyte migration and proliferation.** Wound-induced hypoxia in the wound edge stimulates the activation of p38/MAPK in keratinocytes, i.e., increases in p38 phosphorylation. The activated p38/MAPK promotes the phosphorylation of MAP4 and, sequentially, the depolymerization of MTs, essential components of the cytoskeleton in the control of cell migration and proliferation.

To enhance the wound healing process, it is essential to understand the molecular mechanisms underlying the steps responsible for wound repair. MAP4 was primarily identified in mouse neuroblastoma cells in 1984 31. It has been shown that the balance between MAP4 phosphorylation and dephosphorylation controls MT assembly and stabilization. Once phosphorylated, MAP4 can dissociate from MTs, leading to MT disruption 11. However, few observations have correlated MAP4 phosphorylation with epidermal wound healing. In this study, we demonstrated a novel role of MAP4 phosphorylation in wound healing: the hypoxic environment induced MAP4 phosphorylation by activating p38/MAPK signaling in wounded epidermal keratinocytes, which induced MT disassembly and sequentially a marked increase in keratinocyte proliferation and migration (Fig. 7).

The limited oxygen supply and excessive oxygen consumption after acute injury leads to a hypoxic microenvironment in the wounded tissue 32, 33. Hypoxic stress post wounding stimulates gene expression and growth factor synthesis that contribute to wound repair 34, 35. In the present study, we found that hypoxia (2% O2) induced MAP4 phosphorylation and promoted keratinocyte migration and proliferation, while MAP4(Ala), a constitutively dephosphorylated form of MAP4, could inhibit hypoxia- and KI-stimulated keratinocyte migration and proliferation, as well as MT rearrangement in keratinocytes. These results indicated that hypoxia-regulated MAP4 phosphorylation in keratinocytes was involved in hypoxia-induced cell migration and proliferation.

MTs are dynamic polymers of tubulin and are involved in multiple functions, including vesicle and organelle transport, spindle formation and cell migration. The balance between depolymerization and polymerization in MTs has been attributed to the actions of cellular factors that destabilize MTs, such as Op18, and the opposing action of factors that stabilize MTs, such as MAPs 36. Previously, MAP4 was found to be present in proliferating cells, binding to and stabilizing MTs. Altered activity of MAP4 modulated by phosphorylation contributed to an increase in MT turnover and affected cell cycle progression 37, 38. Additionally, MT disruption facilitated the assembly of focal adhesions and enhanced cell migration 6, 39. Our results demonstrated the promigration and proproliferation effects of MAP4 phosphorylation at sites within the MT-binding domain through regulation of MT dynamics. These results provide support for the role of MT depolymerization in wound healing.

Another important question addresses which signaling pathway is responsible for MAP4 phosphorylation in epidermal keratinocytes under hypoxia. Accordingly, we found that hypoxia activates the p38/MAPK pathway, which is positively related to MAP4 phosphorylation in hypoxic HaCaT cells and primary mouse keratinocytes. In addition, growing evidence indicates that p38/MAPK is an important pathway involved in diverse biological processes under hypoxia 40-42. We used the pharmacological inhibitor SB203580 (5 μM) and the endogenous p38/MAPK activator MKK6(Glu) to suppress or activate p38/MAPK signaling and then observed changes in MAP4 phosphorylation, MT disassembly, cell migration and proliferation in epidermal keratinocytes. We found that activation of p38/MAPK stimulated MAP4 phosphorylation, MT disassembly, cell migration and proliferation in epidermal keratinocytes, while p38/MAPK inhibition decreased them under hypoxic conditions. Moreover, overexpressing MAP4(Ala), a constitutively dephosphorylated form of MAP4, significantly abrogated the effects caused by MKK6(Glu) transfected in HaCaT cells and primary mouse keratinocytes. These observations suggested that the p38/MAPK pathway was both required and sufficient for mediating hypoxia-induced keratinocyte migration and proliferation via MAP4 phosphorylation and MT disassembly under hypoxic conditions.

Together, these data suggest a model that defines a molecular genetic axis in wound healing in which p38/MAPK is required for the induction of MAP4 phosphorylation, which leads to MT disassembly to promote wound healing through a cell migration and proliferation mechanism. Our results show a novel role for MAP4 phosphorylation in skin wound healing and provide a potential target for the development of new treatments for defective wound repair.

## Supplementary Material

Supplementary figures and tables.

## Abbreviations

ANOVA: one-way analysis of variance
DAPI: 4', 6-diamidino-2-phenylindole
EdU: 5-ethynyl-2'-deoxyuridine
HE: hematoxylin and eosin
KI: MAP4 (S737E, S760E and S667A) knock in
MAPKs: mitogen-activated protein kinases
MAPs: microtubule-associated proteins
MAP4: microtubule-associated protein 4
MTs: microtubules
MKs: mouse keratinocytes
PCNA: proliferating cell nuclear antigen
PBS: phosphate buffer saline
WT: wild type
