# Supplementary material for: Microtubule-associated protein 4 phosphorylation regulates epidermal keratinocyte migration and proliferation
Source: Int J Biol Sci. 2019 Jul 24;15(9):1962–76. doi: 10.7150/ijbs.35440 (PMC6743305; doi:10.7150/ijbs.35440)
Supplement: Supplementary file 1 — Supplementary figures and tables. [file ijbsv15p1962s1.pdf]

Supplementary material

Microtubule-associated protein 4 phosphorylation regulates epidermal keratinocyte migration and proliferation

Junhui Zhang[1,2*], Lingfei Li[1,2*], Qiong Zhang[1,2], Wensheng Wang[1,2], Dongxia Zhang[1,2], Jiezhi Jia[1,2], Yanling Lv[1,2], Hongping Yuan[1,2], Huapei Song[1,2], Fei Xiang[1,2], Jiongyu Hu[1,2,3*], Yuesheng Huang[1,2*]

[1]Institute of Burn Research, Southwest Hospital, Third Military Medical University (Army Medical University), Chongqing, China.
[2]State Key Laboratory of Trauma, Burns and Combined Injury, Southwest Hospital, Third Military Medical University (Army Medical University), Chongqing, China.
[3]Endocrinology Department, Southwest Hospital, Third Military Medical University (Army Medical University), Chongqing, China.

[*]**Corresponding authors:**
Yuesheng Huang, Institute of Burn Research, State Key Laboratory of Trauma, Burns and Combined Injury, Southwest Hospital, Third Military Medical University (Army Medical University), Gaotanyan Street, Shapingba District, Chongqing 400038, China. Phone: +86 023 68766023. E-mail: yshuang1958@163.com;
Jiongyu Hu, Endocrinology Department, State Key Laboratory of Trauma, Burns and Combined Injury, Southwest Hospital, Third Military Medical University (Army Medical University), Gaotanyan Street, Shapingba District, Chongqing 400038, China. Phone: +86 023 68773162. E-mail: jiongyu-hu@tmmu.edu.cn.

[*]These authors contributed equally to this work.

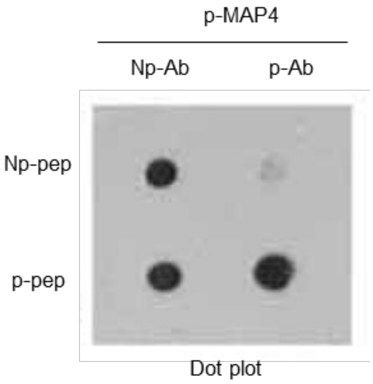

Figure S1. Validation of antibody made in house. Preparation and verification of anti-p-MAP4 polyclonal antibodies by dot blot. Np-pep: non-phospho-peptide, p-pep: phosphor- peptide, Np-Ab: non-phospho-antibody, p-Ab: phosphor-antibody.

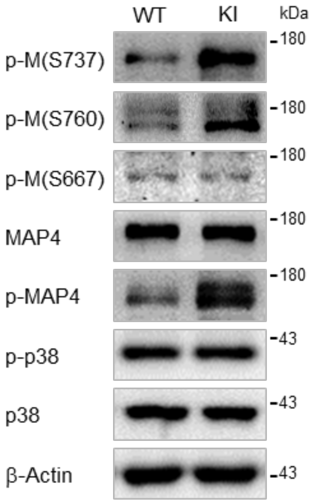

Figure S2. Identification of MAP4 (S667A, S737E and S760E) knockin (KI) mice by Western blot analysis. Western blotting was performed to identify the construction of MAP4 (S667A, S737G and S760G) knockin (KI) mice. β-Actin was used as a loading control.

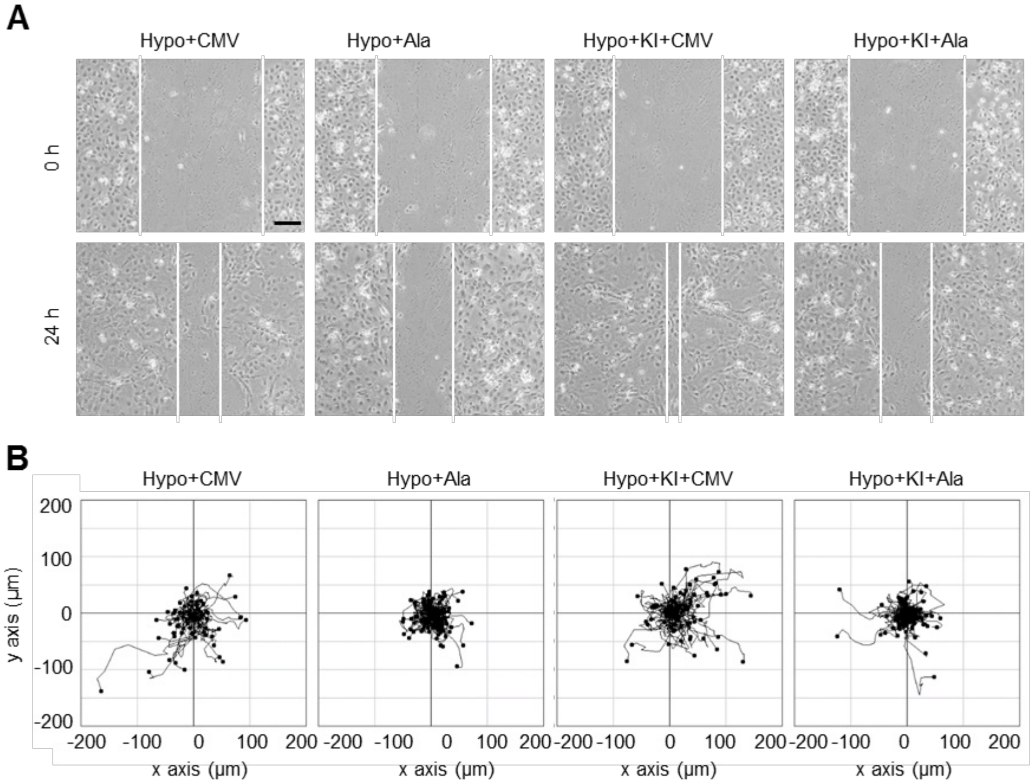

Figure S3. MAP4 phosphorylation regulates hypoxia-induced epidermal keratinocyte migration and proliferation. MKs isolated from the epidermis of KI and WT mice were subjected to hypoxia after being transfected with CMV-null or MAP4(Ala). Keratinocyte migration and motility were assessed by scratch wound healing assays **(A)** and single-cell motility assays **(B)**. Representative pictures of wound healing and the trajectories of keratinocytes are shown. Scale bar = 100 μm. Quantification and statistical analysis are shown in Fig. 4E-F.

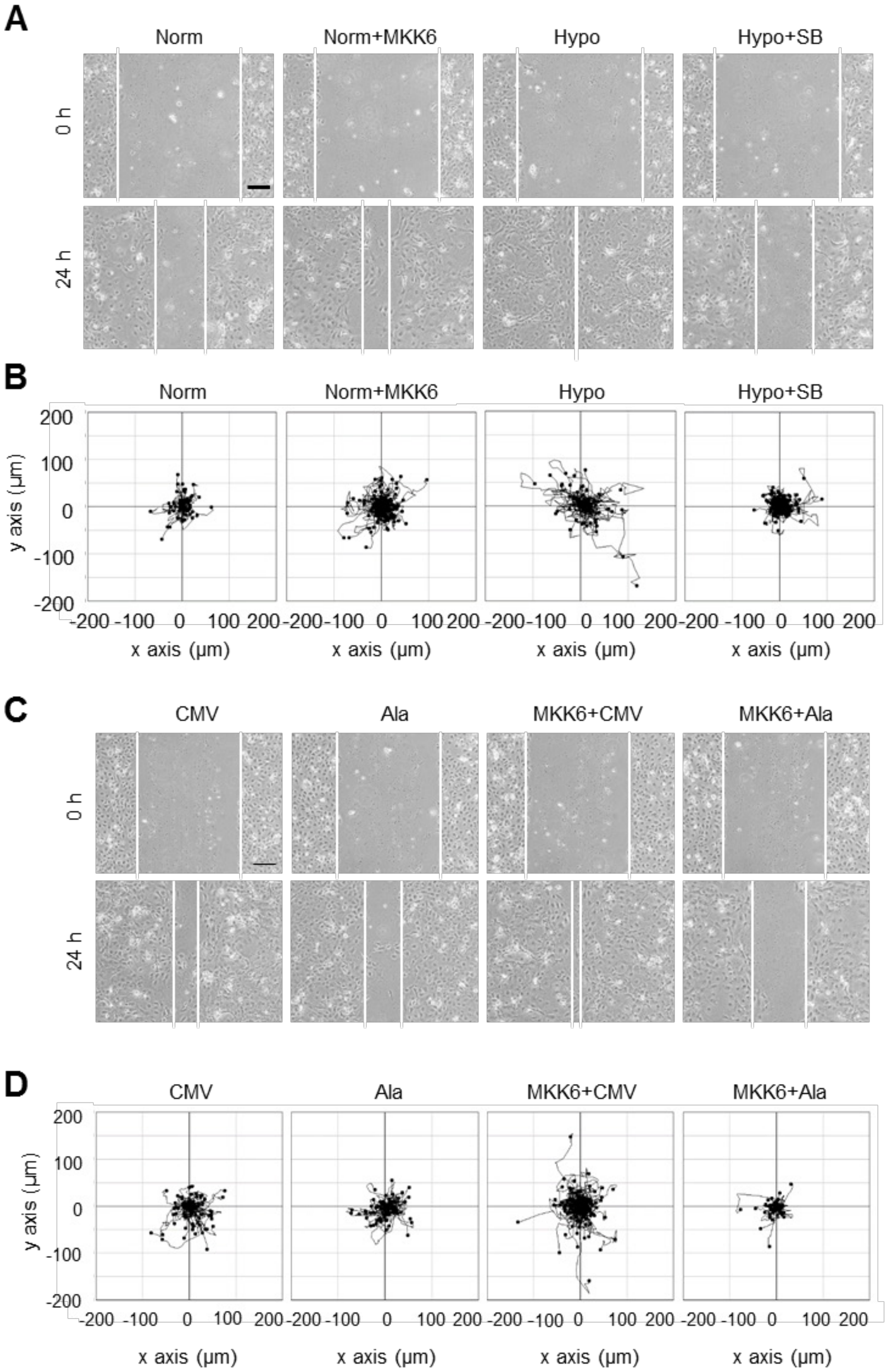

Figure S4. p38/MAPK is involved in MAP4 phosphorylation-induced migration and motility in mouse epidermal keratinocytes under hypoxia. (A-B) MKs were transfected with MKK6(Glu) adenovirus under normoxia or subjected to a specific p38/MAPK inhibitor SB203580 (SB, 5 μM) before hypoxia exposure. Scratch wound healing assays **(A)** and single-cell motility assays **(B)** were performed to determine the migration of indicated keratinocytes. Representative pictures of wound healing and the trajectories of keratinocytes are shown. Scale bar = 100 μm. Quantification and statistical analysis are shown in Figure 5H-I. **(C-D)** MKs were transiently transfected with MAP4(Ala) or MKK6(Glu) adenovirus or both. Scratch wound healing assays **(C)** and single-cell motility assays **(D)** were performed to determine the migration of indicated keratinocytes.

Representative pictures of wound healing and the trajectories of keratinocytes are shown. Scale bar = 100 μm. Quantification and statistical analysis are shown in Figure 5K-L.

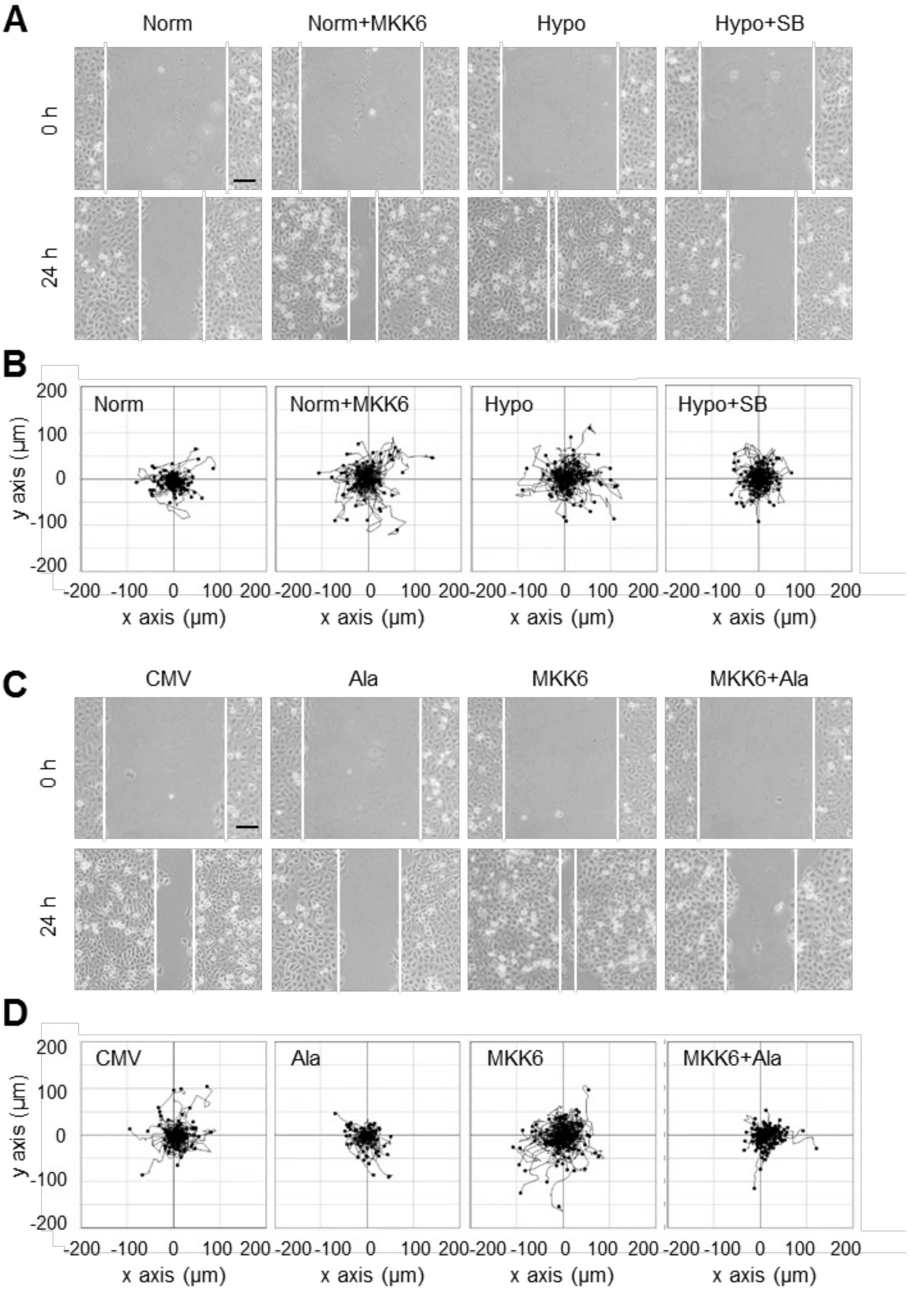

Figure S5. MAP4 phosphorylation is involved in the hypoxia-induced migration of human keratinocytes. HaCaT cells were transfected with MKK6(Glu) adenovirus under normoxia or subjected to SB (5 μM) before hypoxia exposure. Scratch wound healing assays **(A)** and single-cell motility assays **(B)** were performed to determine the migration of indicated keratinocytes. Representative pictures of wound healing and the trajectories of keratinocytes are shown. Scale bar = 100 μm. Quantification and statistical analysis are shown in Figure 6G-H. **(C-D)** HaCaT cells were transiently transfected with MAP4(Ala) or MKK6(Glu) adenovirus or both. Scratch wound healing assays **(C)** and single-cell motility assays **(D)** were performed to determine the migration

of indicated keratinocytes. Representative pictures of wound healing and the trajectories of keratinocytes are shown. Scale bar = 100 μm. Quantification and statistical analysis are shown in Figure 6K-L.

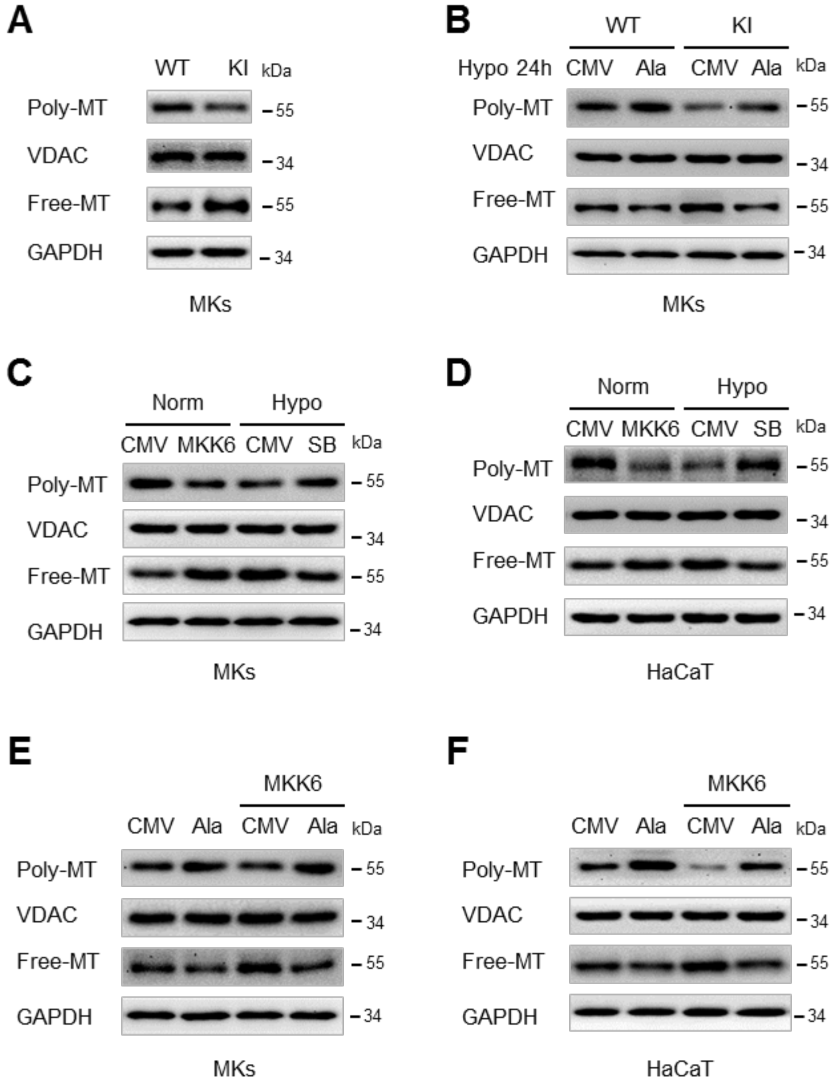

Figure S6. p38/MAPK is involved in MAP4 phosphorylation-induced MT rearrangement in both mouse and human epidermal keratinocytes under hypoxia. (**A and B**) MKs isolated from the epidermis of KI or WT mice were transfected with MAP4(Ala) or CMV-null adenovirus. (**C and D**) MKs (isolated from the epidermis of WT mice) and HaCaT were transfected with MKK6 (Glu) adenovirus under normoxia or subjected to SB (5 μM) before hypoxia exposure. (**E and F**) MKs (isolated from the epidermis of WT mice) and HaCaT were transiently transfected with MAP4(Ala) or MKK6(Glu) adenovirus or both. Western blotting was performed to analyze the polymerized and free tubulin levels; VDAC and GAPDH were used as the loading controls for these fractions (n = 5).
